# Spatial synergies for urban foraging: A South African example

**DOI:** 10.1007/s13280-024-02094-5

**Published:** 2024-11-26

**Authors:** Mallika Sardeshpande, Charlie Shackleton

**Affiliations:** 1 Department of Environmental Science, Rhodes University, Makhanda (Grahamstown), South Africa; 2 University of KwaZulu-Natal, Scottsville, 3209 South Africa

**Keywords:** Collaborative governance, Common pool resources, Landscape design, Urban greenspace, Urban planning

## Abstract

Urban foraging for wild plant and animal products is increasingly recognised for its multiple benefits for people and nature stewardship. Planning for foraging in urban greenspace is one way that foraging can be made more accessible, beneficial, and equitable. Here, we explore how foraging could be recognised and provided for in urban municipalities in eastern South Africa. We synthesise forager and land manager interviews, policy analysis, and iterative discussions to develop four land use configurations under which foraging could be planned for. Design principles for community-based natural resource management are used to assess the feasibility of each configuration. We identify stakeholders who would be involved in governing each configuration, and how urban foraging can co-produce desirable outcomes for their priorities. We list locally-specific actions to enable collaborative greenspace governance through urban foraging. We suggest some generally applicable design considerations and implications for each of the four configurations.

**Supplementary Information:**

The online version contains supplementary material available at 10.1007/s13280-024-02094-5.

## Introduction

Urban foraging, the phenomenon of humans collecting naturally occurring biological resources from urban environments, is gaining traction as an interdisciplinary research area with tangible urban planning and policy implications (Shackleton et al. 2017; Hurley and Emery 2018; Sardeshpande and Shackleton 2020a). Foraging embodies a crucial intersection of the multiple values of nature, especially in urban landscapes where nature and natural agency is constrained (i.e. much of urban nature is human managed). Researchers and practitioners advocate knowledge transfer, inclusive policies, and greenspace provisioning as ways to realise the various benefits of foraging for people and biodiversity (Sardeshpande et al. 2021).

### The contribution of foraging to urban households

Foraged species are used by between three and six billion people globally (Shackleton and De Vos 2022). Urban foraging for wild species may occur within household yards and neighbourhood streets, parks and gardens, as well as fallows, farms, and forests in the vicinity of urban areas (Rupprecht et al. 2015; Russo and Cirella 2019). Studies find that households that forage likely have more diverse sources of food, and consequently, higher dietary diversity (Sardeshpande and Shackleton 2020b; Cheek et al. 2023) than non-foraging households. Foragers often cite the perceived health benefits such as the fresh and nutritious nature of foraged foods as a motivation for foraging for them (Garekae and Shackleton 2020; Rombach and Dean 2023). Indeed, foraged foods can be a source of important and high quality micronutrients, and many of them possess medicinal properties (Broegaard et al. 2017; Bvenura and Sivakumar 2017), leading to their use in traditional healthcare (e.g. Mollee et al. 2017). Savings and sales from foraged materials such as fruits and fuelwood can contribute significant cash income to urban households (Kaoma and Shackleton 2015; Sardeshpande and Shackleton 2023). Besides being a source of nutrition, materials, and income, foraging also forms an important part of natural resource users’ experiences of culture and nature, with a recent synthesis showing that access to wild resources is correlated with improved wellbeing generally, particularly in poorer communities (Wells et al. 2023).

### Foraging at the confluence of culture and conservation

Foraging has significant cultural connotations within forager communities globally (Peckham et al. 2013; Schulp et al. 2014; Hurley et al. 2015; Schunko et al. 2015; Landor-Yamagata et al. 2018). Foragers assert that they value the free and communal access to resources, knowledge transmission through friends and family, social cohesion, lifestyle choices, maintenance of traditional or cultural knowledge, products and processes, and recreational opportunities associated with foraging. Thus, foraging offers people the possibility of maintaining a measure of sovereignty (Grey and Patel 2015; Augstburger et al. 2019), and exercising their traditional and ecological knowledge and management systems (Kremen and Merenlender 2018). These alternative and traditional systems often embed adaptation and resilience strategies by virtue of being practised over generations in their specific local and cultural contexts (Mabhaudhi et al. 2016; Walsh-Dilley et al. 2016). Whereas industrial economies place emphasis on production and consumption, traditional systems tend to incorporate more nuances such as social structures and ecological processes in the procurement of resources (Nyman 2019), of which foraging is an example. These diverse and devolved traditional economies provide alternative pathways for sustainable development, particularly in the Global South, where they persist but risk being replaced by industrial economies (Mabhaudhi et al. 2017; Bergius and Buseth 2019). Efforts to perpetuate traditional economies include the valorisation of underutilised species and practices (de Oliveira Beltrame et al. 2018; Gregory et al. 2019) and the recognition of sense of place and cultural values of biodiversity (Gavin et al. 2018; Brown and Murtha 2019). Foraging is an embodiment of both of these actions, and can be leveraged for the conservation of species, cultures, and landscapes, in their local contexts.

### The interface between foraging and urban biodiversity

Urban foraging involves the collection of cultural, edible, medicinal, and timber products from uncultivated landscapes (Shackleton et al. 2017). Although some foraged species require specific ecological conditions to thrive (e.g. Brokamp et al. 2011; Isaza et al. 2017), many are generalists, and are found growing on fallow or otherwise unproductive land, as well as in heavily used and modified agricultural land and urban areas (Mandle and Ticktin 2013, Lankoande et al. 2017). The ubiquitous habitat range of many foraged species may be attributed to their resilience to environmental pressures, and their dispersal by animal and human users. Some foraged species act as keystone species in their ecosystems, providing food and refuge to wild birds, insects, and mammals, some of which may be charismatic or flagship species themselves (Sardeshpande and Shackleton 2019). Many foraged species nourish and depend upon a diversity of indigenous pollinators and dispersers (Shanley et al. 2012; Venter and Witkowski 2013; Sekar and Sukumar 2015; Shackleton et al. 2018), and particularly in urban ecosystems, can provide habitat as well as corridors for wildlife as a conduit between greenspaces (Champness et al. 2019; Zietsman et al. 2019). In addition to supporting other biodiversity, some foraged species represent important genetic resources that can be cultivated as resilient crops in a changing climate (Leakey 2020). Promoting foraging by humans involves estimating and communicating sustainable yield thresholds and harvesting practices (e.g. non-destructive extraction at the appropriate time of year), in order to ensure plant and population vitality and ecological function. Some of this knowledge already exists in foraging communities (e.g. Thomas et al. 2017), and in some cases, further research is required.

### Foraging as a form of urban landscape stewardship

In addition to its relation to provisioning, cultural, and supporting ecosystem services, foraging also allows urban residents to actively manage their natural resources and regulate ecosystem function to some degree. For example, some exotic species, which are common in managed urban greenspace (Nielsen et al. 2014; Champness et al. 2019) can turn invasive in unmanaged greenspace. Removal of invasives and propagation of indigenous species may offer benefits such as carbon and water sequestration, nutrient cycling, and soil stabilisation (Palliwoda et al. 2017; Synk et al. 2017). Co-management through collaborative arrangements between foragers, institutions, and other stakeholders can enable better locally tailored, low-cost outcomes for urban greenspace (Buijs et al. 2019). Foraging as a form of landscape stewardship will require strategic partnerships across different departments and systematic planning to make urban green infrastructure multifunctional (Artmann et al. 2019; Hansen et al. 2019). Promoting sustainable foraging in public spaces will call for specific policy framings and actions from different stakeholders, particularly the local government, to institute tenure rights, devolved governance systems, and collaborations for co-management of foraging spaces and species with different actors. Formalising the otherwise casual practice of foraging through regulations is likely to allow for the dissemination and enforcement of sustainable harvesting practices, and afford stewardship and protection to the open spaces in which foraging occurs. However, formalisation would have to be inclusive across actors and sectors. This article attempts to envisage the social, ecological, and governance configurations under which such formalisation could be implemented. We define governance configurations as the collection of actors, discourses, networks, processes, and rules that effect governance, in this case, of urban greenspace (e.g. Vaas et al. 2017; Baud et al. 2021; Sekulova and Mallen 2024).

This article approaches the pathway of greenspace provisioning and its possible configurations in the context of South African cities. We assess the current social-ecological and urban planning and policy landscape and ask: What is the feasibility of promoting foraging across different types of urban greenspace? We use common pool resource theory (Ostrom 2009) as a theoretical lens to evaluate different types of urban greenspace as common pool resource systems. We use local data about the existing state of greenspace and extent of foraging therein, the scope for education and planting of species to enable foraging, and the institutional arrangements relating to the use and management of greenspace. We synthesise four possible foraging greenspace configurations, and identify the policy and practice opportunities and constraints, and key stakeholders involved. We assess these four potential configurations against the design principles for community-based natural resource management (Cox et al. 2010), a subset of common pool resource theory. Lastly, we list specific actions for stakeholders to further their agendas of sustainable foraging (common pool resource use) and urban greenspace (common pool resource systems) management through partnerships with urban foragers.

## The local context

South Africa is characterised by legacy spatial planning and in some cases multiple land governance systems. Rapid urbanisation across metropolitan, small and medium towns is resulting in urban sprawl and informal settlements with poor living conditions (Oranje et al. 2020). Racial segregation until the early 1990s resulted in significant socioeconomic inequality (Noble and Wright 2013, StatsSA 2017). Spatial segregation has also constrained the access of poorer communities to economic opportunities, including land-based livelihoods. The post-apartheid government’s Reconstruction and Development Programme (RDP) aimed to redress this by providing housing and services to historically disenfranchised people. However, the effort to improve living standards still falls short of providing equitable access to greenspace and its ecosystem services to many (Venter et al. 2020). Traditional governance regimes may further compound this issue by misappropriation of communal land for private enterprise and illegal development (Olofsson 2021; Lidzhegu and Kabanda 2022). Notwithstanding, the need for improved urban greenspace provisioning presents an opportunity for planning and planting with a focus on urban foraging. Indeed, the presence of devolved land tenure systems provides avenues for greater engagement and localised governance platforms. The presence of explicit and inclusive urban spatial development plans and policy (e.g. Quayle and Pringle 2013; Umhlathuze 2018; Ethekwini website 2019) allow inroads to recognising foraging as a legitimate land use that is planned for. This is especially applicable in South Africa, which is the pioneer in the African context for protecting and prioritising urban biodiversity and ecosystem services (Guneralp et al. 2017).

South Africa boasts nine biomes, many with high numbers of endemic species (Mucina and Rutherford 2006). Biodiversity management strategies span the spectrum of invasive alien control and restoration, land zoning, payments for ecosystem services, and sustainable bio-economy initiatives. There is emphasis on restricting exotic species, and planting of locally specific indigenous species where greening and restoration is undertaken (van Wilgen and Wannaburgh 2016). Therefore, this article focuses mainly on the planting of indigenous species that are known to be used by foragers. The study was conducted in the Indian Ocean Coastal Belt, which is host to much of the woody perennial biodiversity in South Africa, and is among the most rapidly urbanising biomes (Jewitt et al. 2015). Hence, the foraged species considered here are mainly trees; while this choice of plants can be extrapolated to other biomes, we acknowledge that greening for foraging may manifest in other opportunities and constraints in the form of non-tree growth forms such as grasses, herbs, shrubs, succulents, etc. Indigenous trees are often resilient to local ecological stresses and shocks and disturbance (Gaoue et al. 2016; Leakey 2018). In urban areas, they can provide habitat connectivity for wildlife, including pollinators that are important to rural and urban food production (Bennett and Lovell 2019). This makes them ideal candidates for planting for foraging in urban areas which are fragmented landscapes of high-intensity human use.

## Research design

This article synthesises qualitative data collected in nine urban municipalities along the eastern coast of South Africa, as part of studies by Sardeshpande and Shackleton (2020a, b, 2023). The data includes semi-structured interviews with urban foragers about their foraging practices, barriers, enablers and aspirations, and semi-structured interviews with municipal officials involved in greenspace management about their perspectives on urban foraging in the greenspaces they manage. The synthesis question is: what are the managed landscapes in which foraging occurs, and how can foragers and managers collaborate for improved governance of these landscapes? The four landscape configurations emerging from the synthesis were discussed in focus groups with municipal greenspace managers to add descriptive depth to the logical and normative implications for governance. These descriptions are transposed against the theoretical framing of design principles for community-based natural resource management (Cox et al. 2010), which is a well-documented form of collaborative governance.

### Data collection

**Fig. 1:**
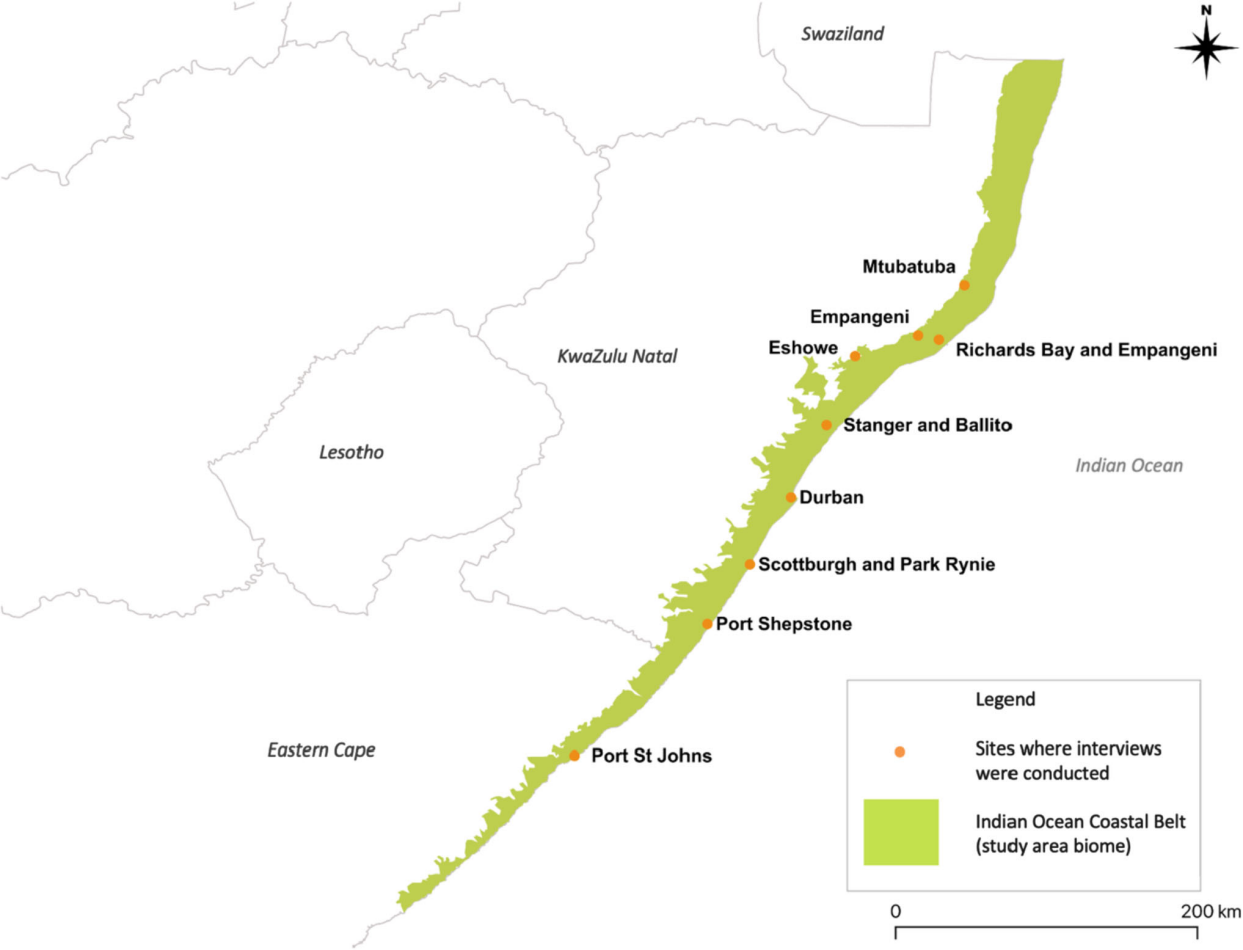
Map of east coast of South Africa locating the study sites within their biome

The data used to synthesise the four configurations were collected between September 2018 and November 2019 from nine urban municipalities in the Indian Ocean Coastal Belt region of South Africa (Fig. 1). Methods included semi-structured interviews with 80 urban foragers (Sardeshpande and Shackleton 2023), and with 15 municipal officials involved in urban greenspace management (Sardeshpande and Shackleton 2020a, b) in the nine municipalities in the region; document analyses of local municipal spatial development plans and policies in these nine municipalities recommended by the interviewed municipal officials; and one focus group discussion each with three officials in two of the aforementioned municipalities (Appendix S1, Sardeshpande 2020). Sampling for municipal official interviews and focus group discussions was purposive, as departments of community services, environmental planning, and parks and gardens were approached and participated in both processes. The methods were approved by the Rhodes University Dept of Environmental Science ethics committee (No. ES17/46, 2017). All participants were provided a brief description of the study, were asked for verbal consent for their interviews to be recorded as audio files, and anonymised, analysed, and presented publicly as research findings.

### Data synthesis and feedback

**Fig. 2:**
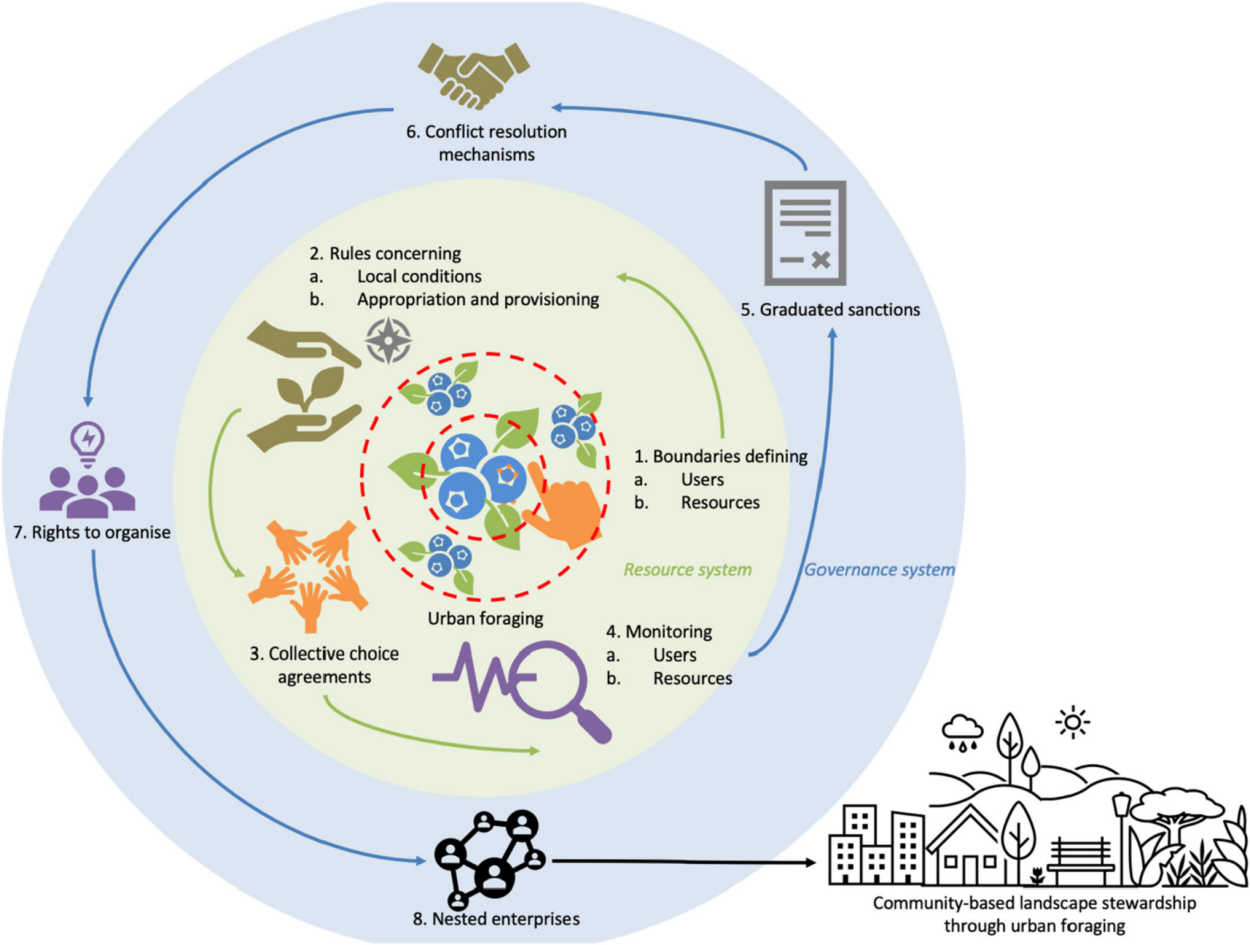
Theoretical mapping of urban foraging as a community-based natural resource management and landscape stewardship strategy

**Fig. 3:**
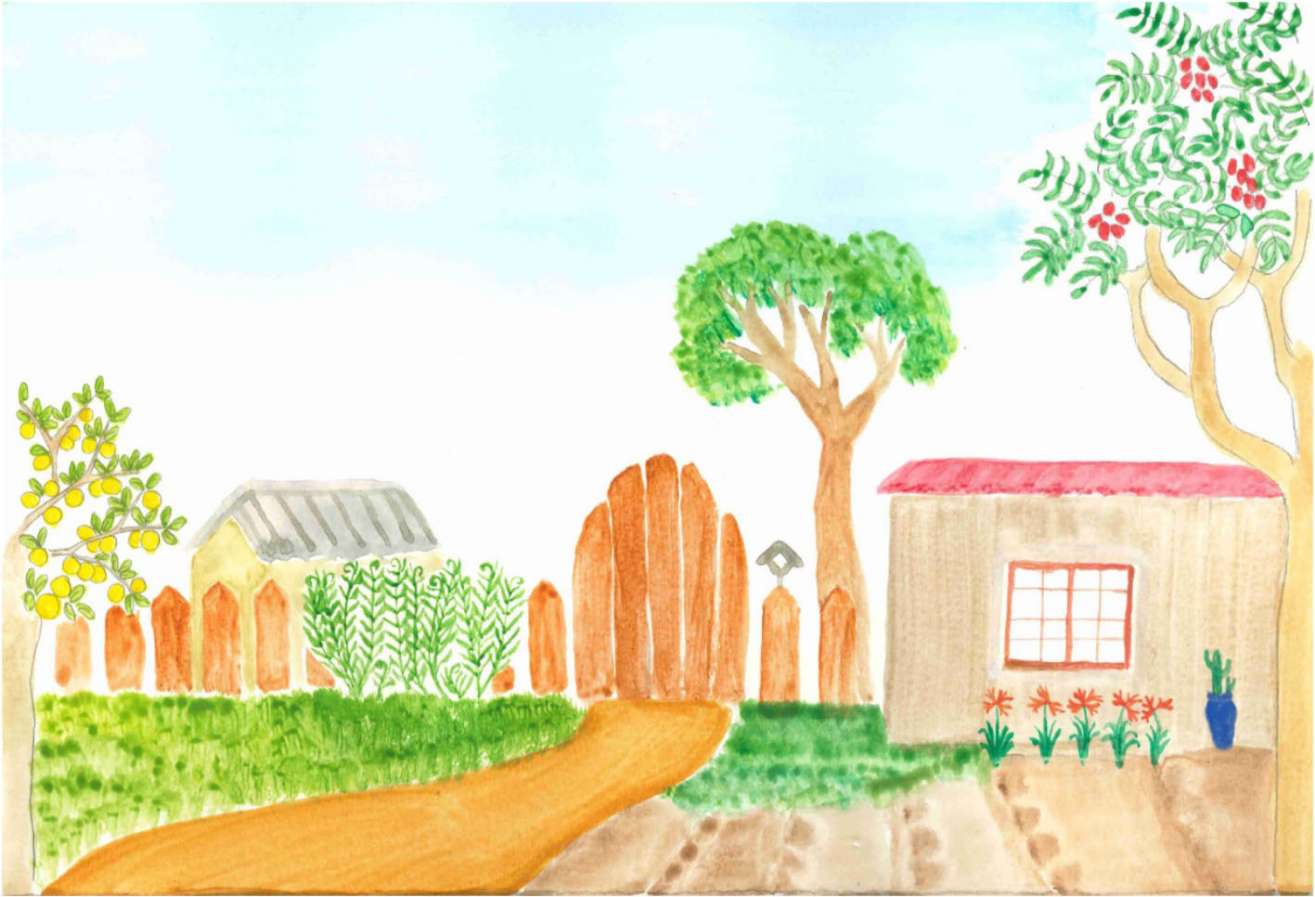
Planting forageable species in residential areas can provide clear boundaries for resource and user access, benefit distribution, and governance, but has low landscape stewardship potential

**Fig. 4:**
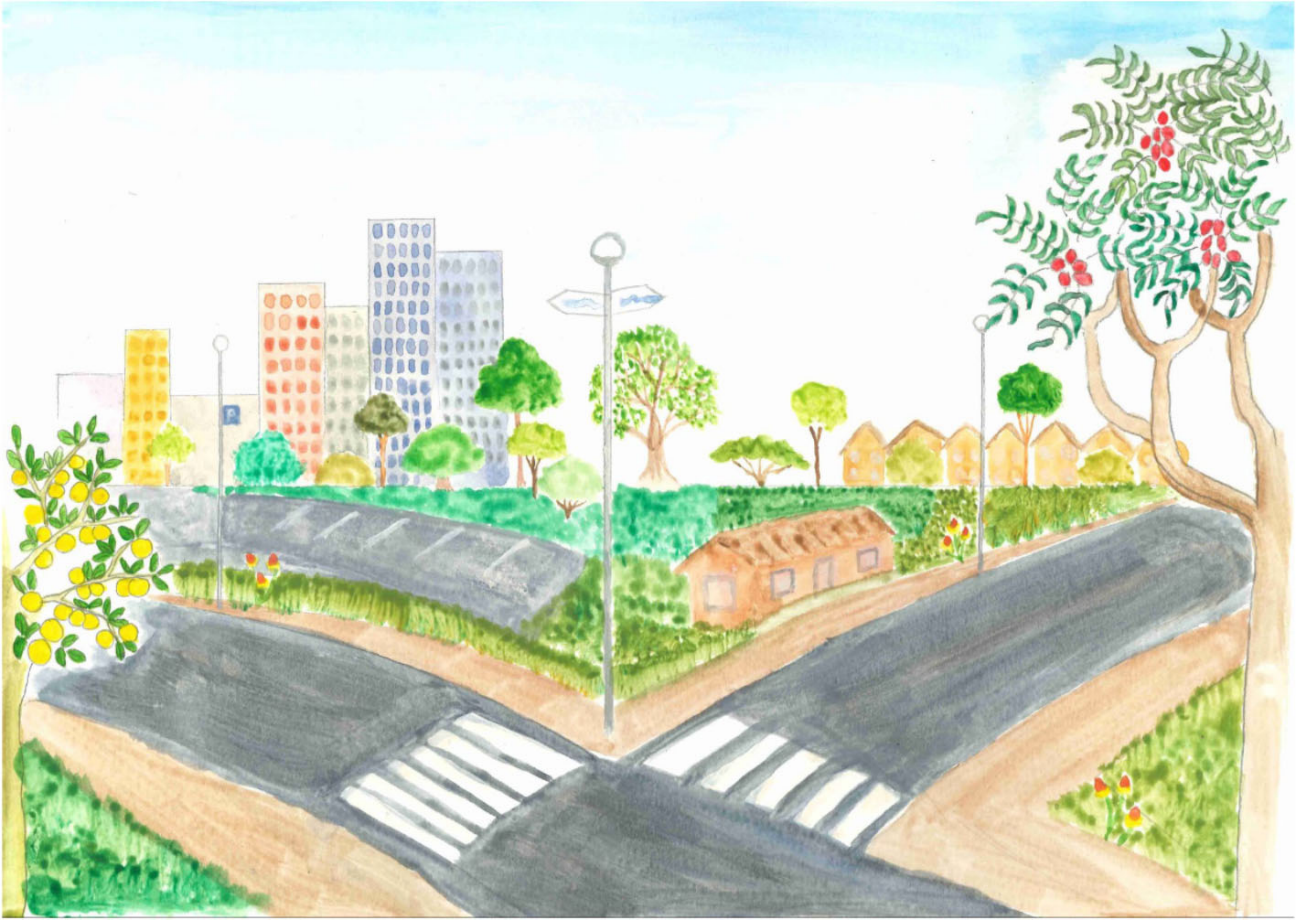
Planting for foraging in peripheral greenspace creates open resource access and involves multiple stakeholders, but can pose risks to benefit distribution and user accountability

**Fig. 5:**
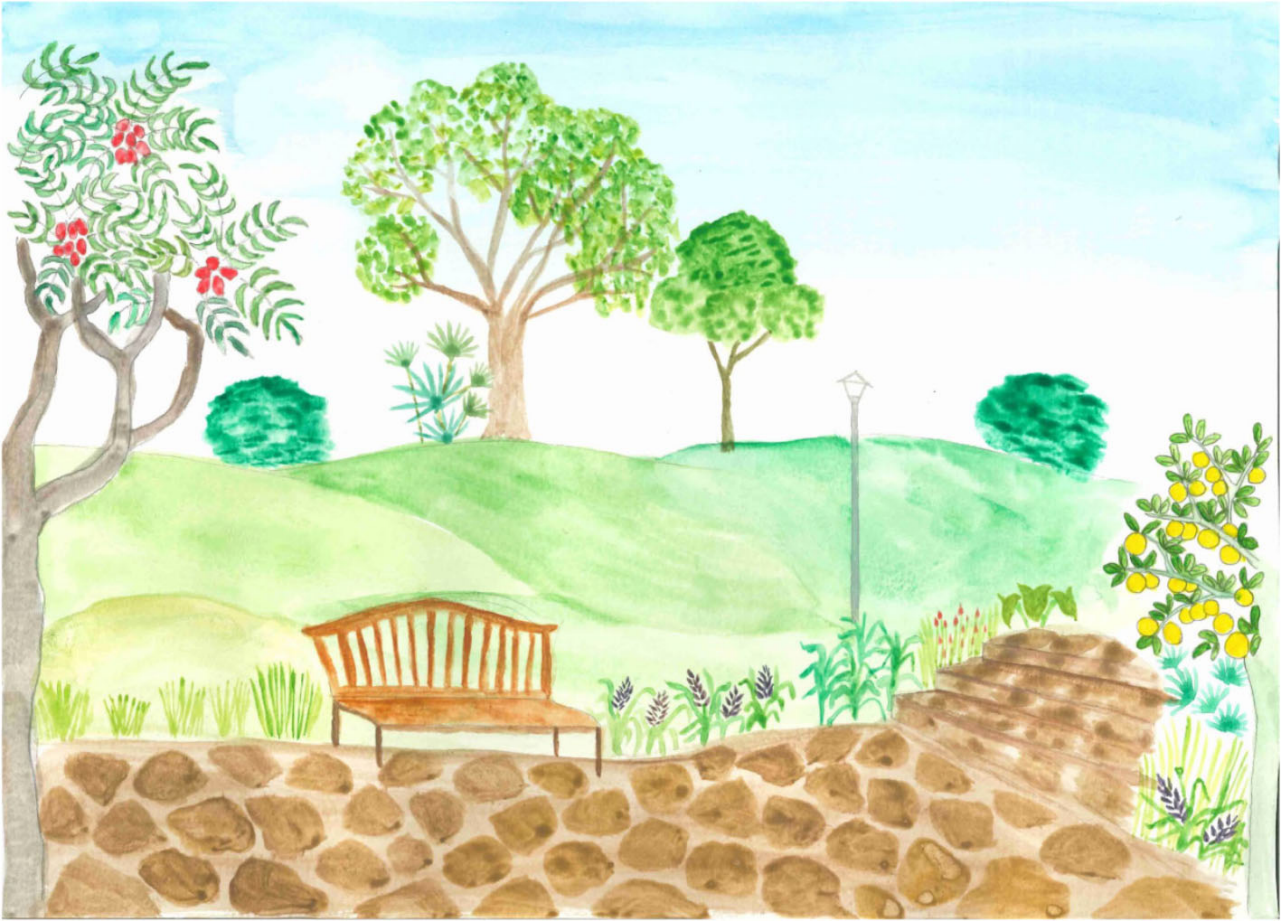
Planting for foraging in dedicated gardens can facilitate systematic access, benefit distribution, and governance, sometimes at the expense of aesthetic values of greenspace

**Fig. 6:**
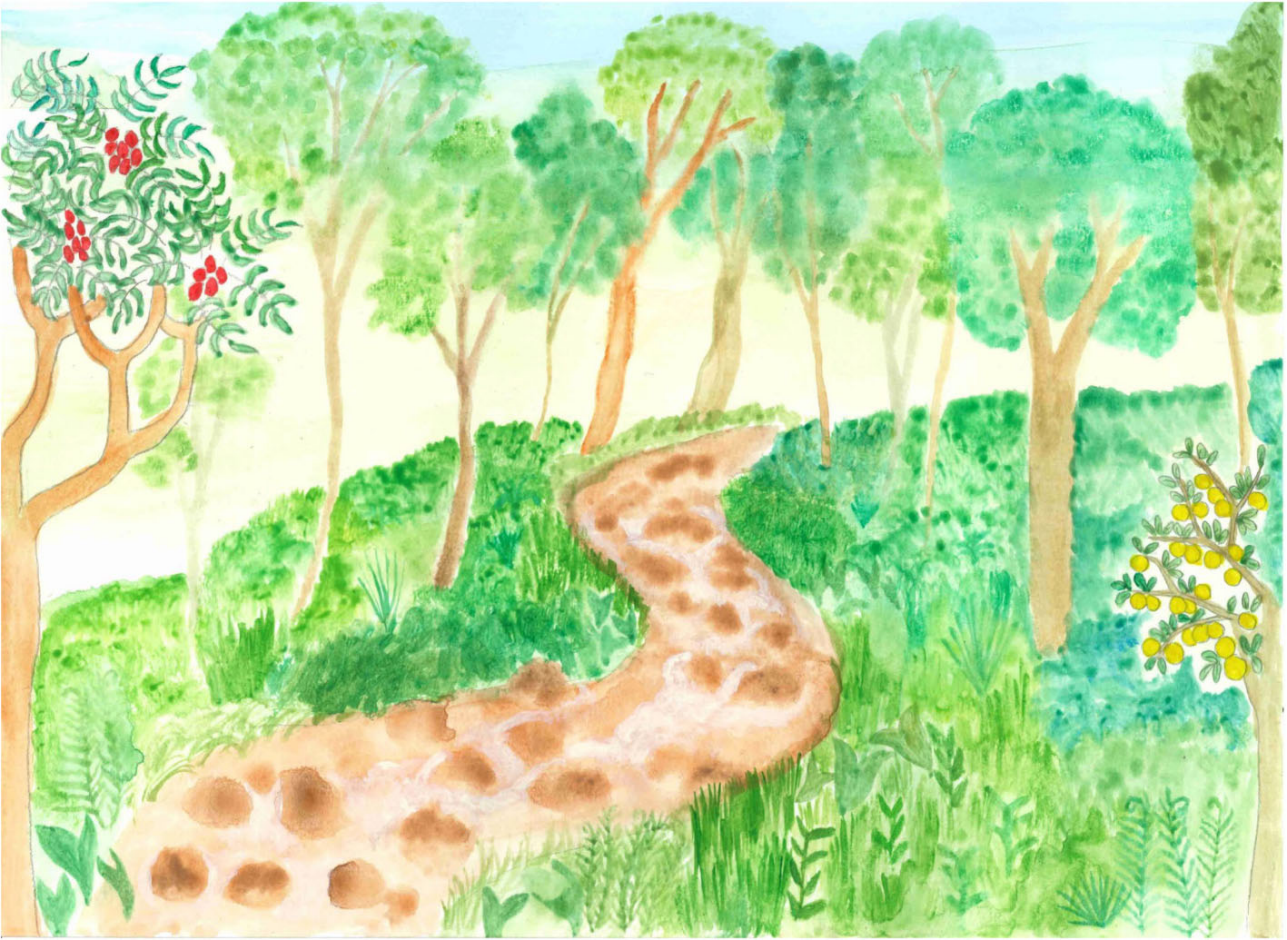
Planting for foraging in natural conservation areas may complement or compromise land management mandates on land use for sustainable livelihoods

Following interview data and document analyses, four possible configurations of foraging in different land use categories emerged. The first author conducted focus group discussions with municipal officials to evaluate the compatibility of foraging with these different urban land uses. Municipal officials were asked to rank the four configurations from least to most desirable, and to detail the execution and management implications of promoting foraging in these categories (Results and Discussion, Appendix S1). Then, the authors mapped these configurations onto the theoretical framework of the design principles for community-based natural resource management (Cox et al. 2010). The configurations were compared for their feasibility against these design principles, and potential impact on common pool resource governance. This article presents the synthesis of the focus group discussions and theoretical evaluation of the foraging configurations. Figure 2 presents the theoretical implications of foraging for community-based natural resource management. Figures 3, 4, 5 and 6 are artistic impressions of the four configurations described in the text. Figures 3, 4, 5 and 6 are artistic impressions by the first author, and none of these figures were included in the data collection or feedback processes; they are presented as a synthesis of the data.

## Theoretical framing: Collaborative conservation of common pool resources

Common pool resource (CPR) theory was developed by Ostrom (1990) to rebut the premise that openly accessible resources with little or no tenure or regulation are vulnerable to depletion due to overuse and misuse. Such degradation of shared resources due to lack of collective governance was termed the tragedy of the commons (Olson 1965; Hardin 1968)**.** CPR theory responds to this standpoint on the tragedy of the commons by identifying different actors, institutions, and regimes that either have, or can, govern such shared resources for long-term sustainability. One of the most central themes in CPR theory is community-based natural resource management (Cox 2014). Community-based natural resource management through collaborative governance has proven instrumental to the implementation of conservation projects on large scales such as continental and marine protected area networks (Wyborn and Brixler 2013; Gruby and Basurto 2014), the Great Barrier Reef (Evans et al. 2014), large multiple use landscapes of conservation value (Guerrero et al. 2015), and river ecosystems across international borders (Villamayor-Tomas et al. 2016). Collaborative governance is key to the functioning of urban systems where stakeholders with varied interests form institutional arrangements to coexist and flourish. The design principles for community-based natural resource management put forth by Cox et al. (2010) present a useful framework to evaluate the feasibility and sustainability of the four potential foraging configurations. The design principles and their implications for urban foraging are outlined in Fig. 2 and Table 1.

**Table 1:** Design principles for community-based natural resource management (Cox et al. 2010)

Principle	Description	Implications for urban foraging in a given foraging space
1A	User boundaries: Clear boundaries between legitimate users and nonusers must be clearly defined	Legitimate foragers and non-foragers should be clearly defined
1B	Resource boundaries: Clear boundaries are present that define a resource system and separate it from the larger biophysical environment	Clear physical boundaries should define the foraging space and separate it from the larger environment
2A	Congruence with local conditions: Appropriation and provision rules are congruent with local social and environmental conditions	Rules about who appropriates (takes, forages) and who provides (resources, labour) should be in line with local social-ecological conditions
2B	Appropriation and provision: The benefits obtained by users from a common pool resource (CPR), as determined by appropriation rules, are proportional to the amount of inputs required in the form of labour, material, or money, as determined by provision rules	The benefits obtained by foragers from the foraged resources and by land managers from foraging services, in accordance with local forager and land rules, should be proportional to the amounts of labour, materials, and money invested by the foragers and the land managers
3	Collective-choice arrangements: Most individuals affected by the operational rules can participate in modifying the operational rules	Most foragers, land managers, and other stakeholders affected by foraging should be able to participate in modifying the rules
4A	Monitoring users: Monitors who are accountable to the users monitor the appropriation and provision levels of the users	Monitors who are accountable to foragers and land managers should monitor the foraging and provisioning levels of the foragers and the land managers
4B	Monitoring the resource: Monitors who are accountable to the users monitor the condition of the resource	Monitors who are accountable to foragers and land managers should monitor the condition of the foraging space
5	Graduated sanctions: Appropriators who violate operational rules are likely to be assessed graduated sanctions (depending on the seriousness and the context of the offense) by other appropriators, by officials accountable to the appropriators, or by both	Foragers who violate rules are likely to be sanctioned (depending on the offense) by other foragers, by land managers accountable to the foragers, or by both
6	Conflict-resolution mechanisms: Appropriators and their officials have rapid access to low-cost local arenas to resolve conflicts among appropriators or between appropriators and officials	Foragers and land managers should have easy access to dialogue and platforms to resolve conflict among foragers or between foragers and land managers
7	Minimal recognition of rights to organise: The rights of appropriators to devise their own institutions are not challenged by external governmental authorities	The rights of foragers to form their own institutions are not challenged by government authorities
8	Nested enterprises: Appropriation, provision, monitoring, enforcement, conflict resolution, and governance activities are organised in multiple layers of nested enterprises	Foraging, investing labour and resources, monitoring, conflict resolution, and governance activities should be organised in multiple layers of interrelated productive activity

The eight design principles for community-based natural resource management (Cox et al. 2010) can be applied to urban foraging as:

- 1A User boundaries: Legitimate foragers and non-foragers should be clearly defined.
- 1B Resource boundaries: Clear physical boundaries should define the foraging space and separate it from the larger environment.
- 2A Congruence with local conditions: Rules about who appropriates (takes, forages) and who provides (resources, labour) should be in line with local social-ecological conditions.
- 2B: Appropriation and provision: The benefits obtained by foragers from the foraged resources and by land managers from foraging services, in accordance with local forager and land rules, should be proportional to the amounts of labour, materials, and money invested by the foragers and the land managers.
- 3 Collective-choice agreements: Most foragers, land managers, and other stakeholders affected by foraging should be able to participate in modifying the rules.
- 4A Monitoring users: Monitors who are accountable to foragers and land managers should monitor the foraging and provisioning levels of the foragers and the land managers.
- 4B Monitoring the resource: Monitors who are accountable to foragers and land managers should monitor the condition of the foraging space.
- 5 Graduated sanctions: Foragers who violate rules are likely to be sanctioned (depending on the offense) by other foragers, by land managers accountable to the foragers, or by both.
- 6 Conflict-resolution mechanisms: Foragers and land managers should have easy access to dialogue and platforms to resolve conflict among foragers or between foragers and land managers.
- 7 Minimal recognition of rights to organise: The rights of foragers to form their own institutions are not challenged by government authorities.
- 8 Nested enterprises: Foraging, investing labour and resources, monitoring, conflict resolution, and governance activities should be organised in multiple layers of interrelated productive activity.

Design principles 7 (minimum recognition of rights to organise) and 8 (nested enterprises) open avenues for devolved, polycentric, and collaborative governance. Collaborative governance implicitly requires the alignment of shared motivations among stakeholders, and a level of trust between them (Emerson et al. 2012; Borg et al. 2015; Walsh et al. 2015). The process of learning through exchange of information and application of different types of knowledge is a key component of collaborative governance (Lauber et al. 2011; Cheng and Sturtevant 2012). Wyborn (2015) proposes a framework for co-production of governance through the iteration and interaction of cognitive, material, social, and normative knowledge. In this analysis, we identify the first three types of knowledge, namely: what the foragers and municipal officials know about foraging in the area (cognitive); what other land uses coexist with foraging in the area (material); what use and governance mechanisms operate in the area (social); to generate the fourth type of knowledge, which is: how these use and governance mechanisms can collaboratively contribute to achieving common goals of sustainable foraging and land use (normative). The scope of this article allows for an iterative process only, and not for analyses of the interactions with power relations that may be inherent in, or may arise from, such co-production of governance (e.g. Fischer et al. 2015; Goodwin 2019). The mapping of local boundaries, systems, arrangements, and actors in relation to this theoretical framework is presented in Table 2.

**Table 2:** Summary and comparison of the four configurations, with respect to the community-based natural resource management design principles, common pool resource outcomes, and key stakeholders involved in maintaining resource systems

Factors	Planting in:	Homes	Verges	Gardens	Reserves
Community-based natural resource management design principles	User boundaries	Clear	Unclear	Variable	Clear
	Resource boundaries	Clear	Unclear	Clear	Variable
	Congruence with local conditions	Not applicable	Possible	Likely to be upheld	Possible
	Appropriation and provision	Possible	Difficult to regulate	Likely to be upheld	Difficult to regulate
	Collective-choice arrangements	Not applicable	Possible	Likely to be upheld	May be compromised
	Monitoring users	Not applicable	Difficult to regulate	Possible	Difficult to regulate
	Monitoring the resource	Possible	Possible	Possible	Possible
	Graduated sanctions	Not applicable	Difficult to enforce	Likely to be upheld	Possible
	Conflict-resolution mechanisms	Not applicable	Difficult to regulate	Likely to be upheld	Difficult to regulate
	Minimal recognition of rights to organise	Not applicable	Possible	Likely to be upheld	May be compromised
	Nested enterprises	Not applicable	Likely to be upheld	Likely to be upheld	Possible
Configuration outcomes for common pool resource management	Access	Undisputed	Public, disputable	Public, disputable	Restricted
	Governance	Private	Municipal	Municipal / public	Municipal / provincial
	Ecological sustainability	Likely to be upheld	Difficult to enforce	Possible to enforce	Possible to enforce
	Collaboration	Not applicable	Possible	Possible	Possible
	Livelihood opportunities	May align with land use	May conflict with land use	May align with land use	May conflict with land use
Executing actors maintaining resource systems	Plant supply	Environment, Parks	Parks	Parks	Environment
	Plant and area maintenance	Parks, Citizens	Parks, Community Services, Citizens	Parks, Citizens	Environment
	Governance	Citizens	Parks, Community Services, Citizens	Parks, Citizens	Environment, Citizens

## Results and discussion

In this section, we describe the four configurations of foraging greenspace design and governance emerging from the focus group discussions. We also integrate examples and learnings from the wider literature where applicable. The configurations are compared for their implications on the design principles of community-based natural resource management, outcomes for people and nature, and the entities involved in land management ("Comparison of configurations for community-based natural resource management", Table 2). We summarise the potential synergies of such collaborative governance with current priorities and commitments on sustainable development, environmental management, and livelihoods ("Harnessing synergies with current priorities and commitments for collaborative governance"). Drawing on the local specificities, we make recommendations to bridge evidence, policy, and practice in the South African context ("Further research and development"). We conclude with wider recommendations and observations for urban foraging as a collaborative governance component for land management in general ("Policy implications").

### Planting indigenous trees in residential areas (yards, complexes)

In some small and medium-sized municipalities (but not metropolitan municipalities like Durban), different households under the RDP receive one (domesticated) fruit tree sapling and one indigenous tree sapling per local household during Arbour Week, which is observed annually in September (DFFE 2022). These saplings are supplied to the households by the Community Services or Environment departments of the local municipality. The procurement of these saplings usually involves recommendations, sponsorships, or budget allotments from the provincial offices of the former Department of Agriculture, Fisheries, and Forestry (DAFF) (now Department of Forestry, Fisheries, and Environment), and the Department of Economic Development, Tourism, and Environmental Affairs (EDTEA). The DAFF, EDTEA, and local municipalities could begin providing ‘forageable’ indigenous tree saplings—with food, fibre, fuel, or medicinal uses—to households. In all municipalities, developers of new and upcoming private housing schemes seek clearance and advice from the local municipality on the greening of their premises. Such developers can also be similarly advised by the Community Services or Environment departments to use forageable tree species. These saplings will mature and bear fruit within five to ten years, and can provide small poles for domestic use such as carving or fencing.

The potential barrier to the adoption of forageable tree species is the public perception of fruit and leaf litter being ‘messy’ and difficult to maintain (see also Shackleton and Mograbi 2020). In the past, trees within household yards have been felled by homeowners to reduce maintenance and litter removal efforts. In some cases, the lack of space in RDP developments has constrained tree planting (similar to Gwedla and Shackleton 2015), and in yet others, preference for backyard rentals has overridden greening efforts (Lategan and Cilliers 2016). In addition to social considerations are physical characteristics of vegetation. Roots and branches of some trees may grow aggressively and damage water or electrical supply lines. Therefore, the species selected for planting in residential areas have to be relatively low-maintenance and low-shedding, small to medium-sized, with docile, compact root and branch growth to facilitate uptake by urban homeowners. The foreseeable benefits of planting forageable trees in residential areas include the provision of undisputed and well-defined access to household or local residents, thereby reducing the risk of conflicts of resource allocation. This is also perceived as a way of ensuring sustainable and steady supply of foraged resources to residents in contrast to unpredictable and variable supply from untended open spaces.

Planting of forageable tree species in residential areas will aid the stewardship of land in private or communal ownership, and provide easy access to and reliable supply of foraged resources. However, it will not contribute to landscape or ecosystem-level management and stewardship, nor will it promote multi-stakeholder cooperation. Many foragers rate the recreational value of foraging highly (Sardeshpande and Shackleton 2023), and planting forageable species within residential areas will provide limited opportunities for landscape-level recreational activities associated with foraging. Nevertheless, this configuration is favourable for the promotion and propagation of the culture and knowledge of alternative and traditional indigenous foods, medicines, crafts, etc., by making these species that are underrepresented in urban settings more accessible to households. Planting for foraging in residential areas is a feasible configuration for small and medium-sized municipalities, with the Community Services and Parks departments, respectively, being the main actors in executing the necessary actions. It has limited potential in the metropolitan municipality of Durban, where housing development occurs at high intensities, and residents’ involvement with foraging spaces would be constrained.

### Foraging in peripheral green infrastructure (verges, edges)

Many foragers pick wild edible fruits and herbs and medicinal species from the edges of roads and sidewalks, often when walking to work or neighbourhood destinations. However, in the lists of plant species procured and planted by the local municipalities, only three species of wild edible fruits were found (*Diospyros whyteana*, *Harpephyllum caffum*, *Trichilia dredgeana*). Inferentially, the trees from which wild edible fruits are foraged were planted in the past, by private or non-municipality planters, or grew there incidentally. The DAFF, EDTEA, and local municipalities could therefore include more species of foraging importance in their plantings in these spaces. The premises of public offices such as police stations and schools were also commonly mentioned as sites where wild edible fruits were foraged. Some municipalities encourage the planting of domesticated food-bearing species (like cabbage and spinach) in peripheral open spaces such as verges and school premises to augment urban and sustainable food production. In such cases, the municipality might supply seeds and seedlings, manure, soil, and helps build citizen capacity in organic intensive farming. Municipalities engaged in such capacity development can potentially include forageable species of herbs and trees in their seeds and seedlings that they supply. Some foragers and municipal officials also suggested planting of forageable trees on the periphery of public spaces such as playgrounds and parks. Indeed, these edges would serve multiple purposes of fencing, shading, and resource provisioning, and in some cases, foragers reported useful trees around playgrounds.

Foreseeable risks in planting for and foraging in peripheral green infrastructure include over-extraction, allocation disputes, logging, and theft (see also Richardson and Shackleton 2014). Free and open access to forageable trees may attract high numbers of foragers or high volumes of harvest (possibly for commercial purposes), affecting the vitality of the trees, and potentially creating high disturbance in their surroundings. In the absence of regulations and ownership, there might arise issues of who forages, and how much is foraged. Trees from peripheral greenspaces are logged in some places, often for fuelwood, and therefore, woody species of foraging importance may be threatened by such use. In some cases, (domesticated) fruit species saplings as well as valuable ornamental species planted by the municipality in peripheral greenspaces have been stolen. With these considerations, the species selected for planting in peripheral greenspace have to be relatively heavy-bearing and resilient to harvest and disturbance. Further, where such species are planted, information and instructions on sustainable seasonal use and harvest precautions will have to be communicated explicitly, either through signage or regular exposure. Local resident associations may volunteer to take responsibility of the forageable trees in their neighbourhood to ensure sustainable harvest and prevent logging or theft. In our experience with urban greening in the biome, trees require up to six months of watering after planting, following which they self-sustain. However, we acknowledge that in other contexts, access, costs, and labour related to watering and plant care may be a key consideration, which could be addressed by selection of low-maintenance species.

Structural and spatial contexts would also play an important role in planting for foraging. Similar to the first configuration, planting along verges of humanmade infrastructure such as walkways and embankments would require the roots to be compact so as to not impact the infrastructure, and the branches to be routinely maintained to prevent physical and visual obstruction, especially along roads. Planting for foraging would be appropriate in areas with relatively low or slow vehicular traffic and high pedestrian traffic, to minimise hazards. Relatively secure and peopled public premises with adult supervision, such as hospitals, police stations and schools, would be suited to planting for foraging, so as to reduce the risk of mishaps related to children and crime, as signage may not be accessible to or heeded by all users. Bearing these specifications, planting for foraging along peripheral green infrastructure is likely to aid collaboration between various stakeholders towards maintaining this infrastructure and augmenting its functionality (Gwedla et al. 2022). Besides providing food, it may also help prevent erosion, provide transit corridors for biodiversity, improve waste management through vigilance, and increase social cohesion (Du Toit et al. 2018).

Planting and foraging in peripheral green infrastructure will ensure free and open access to foraged resources for all. In addition to improving resource availability, it will also increase visibility and subsequently awareness of sustainable foraging. There are however a number of limiting factors from social, structural, and spatial standpoints, which need to be factored into decisions on which species to plant, and where to plant. Such a scenario has the potential to bring together local neighbourhoods for landscape stewardship, not only from a resource management perspective, but also towards better waste and infrastructure management, and potentially neighbourhood and biodiversity monitoring. The key collaborators would be the Community Services and Parks departments in the municipality, managers of public premises, and representatives of the communities where such planting is done. This configuration is feasible in all levels of municipalities, but is likely to be more effective where staff from each stakeholder group can monitor the infrastructure routinely (e.g. Roman et al. 2017).

### Foraging in consolidated and dedicated greenspace (parks, gardens)

Planting for and promoting foraging in designated greenspace, namely parks and gardens, was considered a favourable configuration by foragers as well as greenspace managers. The distinctive feature of such designated spaces was the incorporation of rules and regulations for sustainable and equitable foraging. The knowledge that foraging in such spaces is legitimate, regulated, and encouraged would attract foragers (especially in the study area, see Sardeshpande and Shackleton 2023). Designated areas would ensure that ecosystem disturbances and any potential damage from foraging is contained within the area, rather than distributed across greenspaces of varying degrees of biodiversity value. Important questions raised during preliminary discussions included the lack of information on suitable species, sustainable quantities and methods of harvesting; the potential threat to ecosystems from associated activities such as debarking, logging, and littering; and potential disputes over tenure and allocation. To address these issues, the creation of foraging gardens and parks would require collaborations between multiple stakeholders. Cities that promote citizen-led greenspace governance (Buijs et al. 2019) and public food gardens (Hajzeri and Kwadwo 2019) could prove a useful starting point for the design and development of foraging parks.

While foraging could be encouraged in existing parks and gardens, some land managers argue that extraction can interfere with the aesthetic appeal of spaces designed specifically for recreation. Therefore, a designated area within parks and gardens would be an effective way of containing human disturbance, and also providing sufficient signage and guidance on sustainable thresholds. There is also a common misconception among urban land managers that edible fruit bearing species are rarely indigenous, and that subsequently, planting for foraging may encourage the spread of alien species. The sharing of information on indigenous forageable species during this study has resulted in more interest and willingness to plant these species. There is also the hope that ready accessibility of forageable species in urban greenspace will reduce pressure from human extraction on natural areas intended for conservation, which remains a concern among conservation practitioners in the region (Xaba et al. 2022).

Questions of suitable species and methods and quantities of harvesting can be answered by some research specific to species assessed for their agronomic potential (Nkosi et al. 2020; Dzikiti et al. 2022), or may be initiated based on information gaps identified herein. The design and landscaping of such spaces to optimise efficient production, recreation, and aesthetic value can be aided by the technical expertise of Parks departments. The rules and regulations in dedicated foraging gardens and parks would have to include prohibition of destructive and disruptive practices such as debarking and littering. However, regulations applying to tenure and allocation were not clear, and will have to be discussed with the main stakeholders, namely foragers, greenspace managers (Parks, Environment, and Community Services managers), local residents and associations, and any landowners who agree to lease their land for such a purpose. The Agroecology department of the Durban municipality helps citizens create and operate food gardens or small urban farms in open spaces, and their guidelines for such co-operative farming would be a useful starting point for the formulation of rules and regulations for foraging gardens and parks (Greenberg et al. 2022).

Similar to foraging in peripheral greenspace, foraging in dedicated greenspace would allow for co-management and stewardship of landscapes of provisioning and cultural value in the urban biosphere. It will also increase the accessibility, visibility, and legitimacy of traditional, alternative, and resilient useful species. In contrast with planting in private or peripheral spaces, planting for and foraging in consolidated and designated spaces will necessitate the incorporation of explicit rules and engagement of the community in maintaining these spaces, thereby facilitating information exchanges and building communities of practice. Planting for foraging in dedicated greenspaces is particularly suited to large municipalities like Durban which have distinct greenspaces, often with well-defined mandates, and personnel allocated to their management and maintenance, as well as high through traffic.

### Foraging in conservation areas (reserves, restoration, offset developments)

Planting for and foraging in conservation areas was seen as a possible but not entirely desirable configuration by land managers. Conservation areas constitute different types of natural landscapes that offer varying degrees of protection to the biodiversity they host. Examples of these areas include urban protected areas or parks which preserve relatively untransformed or highly valuable ecosystems and species, where only low impact recreational activities are permitted (Wiggill 2022). Restoration and offset landscapes involve reconstruction or enrichment of specific ecosystems to enhance their functions, or to offset biodiversity impacts from developments elsewhere. In some cases, such conservation areas may be specifically targeted to provide biodiversity-based livelihoods through extractive use of indigenous or alien species. The motive behind all conservation areas is to protect biodiversity and provide ecosystem services, be it cultural and supporting services in protected areas, or provisioning and regulating services in restoration and offset landscapes. Land managers as well as foragers recognised that foraging represents the use of the cultural and provisioning ecosystem services of conservation areas.

Foraging in protected areas could be a potential source of ecosystem disturbance and subsequently degradation, if unregulated. Specifically, activities such as debarking, hunting, littering, logging, and overharvesting may possibly co-occur with extractive use, and would be unfavourable for biodiversity conservation in protected areas. Uninformed or indiscriminate foraging in protected areas may harm both harvested as well as unharvested species, especially vulnerable ones, and may promote the spread of useful alien species such as guava. Further, the possibility of disputes over access to and allocation of resources was also flagged in such a scenario. On the other hand, planting for and promoting foraging in restoration and offset landscapes, particularly those linked to livelihoods, was seen as a favourable configuration by land managers. Planting for and promoting foraging in buffer zones between human habitation and protected areas, including as ‘live fences’ that are productive for both humans and wildlife would be considered feasible. Land managers affirmed that foraging and livelihoods linked to it (such as local craft food supply chains and ecotourism enterprises, e.g. Veld and Sea 2023; Wild Hermanus 2023; Wolfgat 2023) could be incorporated as an additional incentive into conservation areas.

Considerations for this scenario are similar to those for foraging in consolidated greenspaces, namely the constitution of agreements between users regarding foraging access and allocation, and communication of and adherence to sustainable harvest quantities and practices. In the case of setting up commercial foraging-based livelihoods, additional precautions will be required. These could include self-regulatory or third-party standards and certifications to ensure sustainable and responsible harvest and improve visibility and market value of foraged products. The most accessible of such standards is the FairWild certification scheme which has been hitherto applied to rural contexts (Morgan and Timoshyna 2016). Foraging in conservation areas has the potential to both aggravate and ameliorate land use conflict. Planting for foraging in conservation areas has limited potential due to the strict rules and regulations that foraging might contravene. This preference may be a reflection of a certain set of land managers’ worldviews of nature and its conservation, which often idealise pristine nature, and view human activity as detrimental to conservation (Sandbrook et al. 2019). Ultimately, the dichotomy between protection of and production in natural landscapes is often resolved to the scale and context of the area in consideration (Artmann et al. 2019; Mell and Clement 2019).

### Comparison of configurations for community-based natural resource management

The establishment of special parks and gardens for foraging, emerges as the most feasible configuration. This configuration has the most well-defined commons boundaries, incorporates all design principles, and poses very little risk to conservation and development objectives (Table 2). The second most feasible configuration is planting for foraging along verges and other peripheral green infrastructure. Such planting could be well-governed by nested enterprises, but could potentially endanger other species or infrastructure in the landscape. Although planting for foraging in private residential areas involves minimal risk, it is not practicable in all contexts, and has limited potential to contribute to multiple objectives. Planting for foraging in conservation areas has equal opportunities and risks, thus proving to be the least favoured configuration. This outcome also reflects some land managers’ views of conservation goals being compromised by human use of landscapes, and thus, a link between CPR theory and the critique of fortress conservation (Cox et al. 2016; Sardeshpande and Shackleton 2020a). Planting of special parks and gardens for foraging also presents the optimum combination of ease of access, governance, collaboration, and livelihood potential. Overall, the Parks department emerges as the key stakeholder for the planting, maintenance, and governance of the open access configurations.

The configurations developed and discussed in this article do not include foraging in informal urban greenspace such as vacant lots, where a significant amount of foraging does occur (Rupprecht and Byrne 2014; Synk et al. 2017; Charnley et al. 2018; Sardeshpande and Shackleton 2023). We propose that some of these spaces could be accorded the status of foraging parks, and enriched with further planting of species of wild edible fruits as well as edible and medicinal herbs, which are often foraged in conjunction. Indeed, emerging research on informal urban greenspace documents the social and ecological value of these spaces (Engemann et al. 2024) while also recognising that user involvement in design is critical to achieving collaborative governance (Breed et al. 2023). We acknowledge that formalisation may contradict the philosophy of foragers who value the unregulated pursuit of resources in informal settings (Galt et al. 2014; Paddeu 2019; Nyman 2019; Iveson et al. 2019). Further, we focus on design configurations synthesised from a collaborative process where informal greenspace did not emerge as a priority, and therefore do not attempt to prescribe arrangements for such.

### Harnessing synergies with current priorities and commitments for collaborative governance

While the literature evidences the potential use of urban infrastructure to enhance its functionality to achieve multiple goals (Rupprecht et al. 2015; Botzat et al. 2016; Säumel et al. 2016; Buijs et al. 2019; Elands et al. 2019), urban land managers have their reservations about such uses. We acknowledge that while public participation and cross-department collaboration are recommended and aspired to by policymakers, achieving these goals can be arduous due to varied interests and willingness (Molin and van den Bosch 2014; Mathers et al. 2015). This applies to the local context, where silo effects hinder effective collaboration between government departments and institutions (Du Toit et al. 2018; Breed et al. 2023). However, efforts to operationalise foraging spaces can align with governance commitments and programmatic action, thereby offering further scaffolding for nested enterprise. For example, resources for planting and propagating forageable species could be mobilised through programmes supporting national commitments to reforestation (PAGE 2017). Planting forageable species could also form part of invasive plant control and landscape restoration efforts (van Wilgen and Wannenburgh 2016). Recognising public use of and contributions to sustaining forgeable biodiversity and the spaces it occurs in can enable positive urban greenspace stewardship outcomes, including waste and catchment management (Davids et al. 2021). Encouraging sustainable use of forageable species in urban spaces through education and provisioning ties into the South African National Biodiversity Economy Strategy (2015). Popularising forageable species in public spaces would complement government initiatives to improve access to fresh foods to combat malnourishment, such as the Integrated Food Security and Nutrition Programme and the Natural Resources Management Programme (DAFF 2019). At the regional scale, these synergies can help link to SDG 1 on reducing poverty, SDG 2 to promote food and nutritional security, SDG 11 on resilient and sustainable human settlements, SDG 12 on sustainable production and consumption, SDG 13 on climate change adaptation, SDG 15 to protect, restore, and promote sustainable use of terrestrial ecosystems, and SDG 16 on inclusive institutions. Indeed, recent trends indicate that cities across the world are explicitly considering multifunctional biodiversity components in their spatial development plans, of which foraging spaces form a part (van Zyl et al. 2021, O’Neill et al. 2024). Locally specific considerations in this regard include planting appropriate species, mobilising leaders and volunteers from within the community to monitor and report changes, and promoting collaboration between stakeholders.

## Further research and development

Based on our findings, we make the following recommendations towards strengthening synergies between foraging and urban landscape stewardship:

1. Improving access to existing information on the occurrence, ecology, and use of forageable species for foragers and land managers. Specific information for the study area on range, morphology, and uses is available in Sardeshpande and Shackleton (2020b) and is being further developed by the authors. In-depth species profiles are available with the South African National Biodiversity Institute (SANBI, 1).
2. Research on optimal harvest practices and yield of forageable species and dissemination of this information to relevant stakeholders. Various academic articles (e.g. Nkosi et al. 2020; Dzikiti et al. 2022), botanical records, certification standards, and indigenous knowledge describe sustainable harvest practices that can be tested and used for different species depending on their abundance and harvest demand.
3. Establishing partnerships between Parks, Environment, and Community Services departments within municipalities, and where possible, with non-governmental organisations and citizen groups to organise the planting and maintenance of forageable species. Some ongoing agroecology and urban development initiatives (e.g. Peterson 2015, Abalimi Bezekhaya 2023, Wild Harvest 2023, Woza Nami 2023) are working towards incorporating forageable species into their household and community food programmes.
4. Planting of forageable species in household yards, around public infrastructure, and in designated areas where applicable and possible to increase awareness and use. The plants and areas can be monitored for plant survival, yield, vitality, and associated biodiversity. Public infrastructure and designated areas can also be testing grounds for effectiveness and implications of varying degrees of resource access and governance. Urban community forestry may be a viable option in some terrains (Burgdorf et al. 2023).
5. Linking users, relevant departments in the administration, and prospective buyers to develop sustainable supply chains linked to urban foraging. Existing indigenous food value chains (e.g. African Marmalade 2023; The Local Village 2023) could include urban foraged products.

## Policy implications

Across geographies, planting for foraging in private residential spaces is likely to improve local dietary diversity and connection with biocultural diversity (Hendriks et al. 2020; Rombach and Dean 2023). Foraging in private residential areas is also likely to reduce the risk of contamination by environmental pollutants (Stark et al. 2019; Amato-Lourenco et al. 2020). The proliferation of benefits from such planting is limited when considered from a community service standpoint. Moreover, planting in private residential areas may be driven by local preferences for ornamental or low-maintenance plants (Zheng et al. 2022), in contrast with forageable species that may require skilled management.

Verges could make a significant contribution to improving accessibility and stewardship opportunities for foraging, given that they occupy about a third of urban public space (Marshall et al. 2019). Maintenance of verges for foraging may in some cases necessitate greater skilled manpower and budgets, which are often constrained even in the Global North (Hargrave et al. 2024). While public access in this configuration is wide, benefit distribution is likely to be diffuse, and the resource at greater risk of depletion than in other configurations. Selection of resilient species (Sardeshpande et al. 2023) and collective action to monitor their status (Roman et al. 2017) can balance opportunities against risks in this configuration. Citizen monitoring and reporting of the status of these species and spaces can encourage effective local stewardship (Landor-Yamagata et al. 2018).

Developing designated gardens for foraging allows for well-defined user and resource boundaries and functions, and governance mechanisms. However, their design and distribution in urban areas needs to explicitly consider equitable access to all socioeconomic strata (Nesbitt et al. 2019; Vargas-Hernández and Zdunek-Wielgolaska 2021). Such designed spaces should also consider ease of access to pedestrians, safety of social groups (Adeyemi and Shackleton 2023), minimised exposure to waste and pollution (Guenat et al. 2023), and knowledge dissemination on safe and sustainable foraging (Russo and McCarthy 2024).

## Conclusion

In this article, we explore the different possible configurations in which foraging can be actively and intentionally encouraged in urban settings. Enablers, barriers, and outcomes for people, nature, and governance vary across these configurations. Designated spaces for foraging in urban green infrastructure emerge as the optimal space with minimised risk and multiple synergies. Verges and other (managed) peripheral green infrastructure emerges as the second most favourable configuration due to its open access, diffuse benefit distribution, and high maintenance. From a policy perspective, operationalising foraging for collaborative governance would entail explicitly allocating budgets and space for foraging as a land use in urban greenspaces. It would also imply including more forageable species in urban greening inventories; designing and planning either designated greenspaces or sections of public greenspaces for extractive human use; and maintenance and monitoring of these greenspaces for sustainable offtake.

## Supplementary Information

Below is the link to the electronic supplementary material.

Supplementary file1 (PDF 610 KB)

## Footnotes

1 http://pza.sanbi.org/.
