# Supplementary material for: Spatial synergies for urban foraging: A South African example
Source: Ambio. 2024 Nov 26;54(4):714–33. doi: 10.1007/s13280-024-02094-5 (PMC11871281; doi:10.1007/s13280-024-02094-5)
Supplement: Supplementary file 1 — Supplementary file1 (PDF 610 KB) [file 13280_2024_2094_MOESM1_ESM.pdf]

Spatial synergies for urban foraging: a South African example

Supplementary Information

This supplementary information has not been peer reviewed.

Page 1: Focus Group Discussion Guide for Urban Greenspace Planners and Managers (This article)

Page 2: Plain Language Summary of Urban Greenspace Planners and Managers Study (Sardeshpande and Shackleton 2020)
Sardeshpande, M., & Shackleton, C. (2020a). Urban foraging: Land management policy, perspectives, and potential. *PLoS ONE*, *15*(4), e0230693.

Page 3: Plain Language Summary of Urban Foragers Study (Sardeshpande and Shackleton 2023)
Sardeshpande, M., & Shackleton, C. (2023). Fruits of the city: The nature, nurture and future of urban foraging. *People and Nature, 5*(1), 213-227.

In the future (by the year 2040), which of these situations would you most like to see? Please rank the situations from most desirable (1) to least desirable (4).

a. Foraging within the premises of the home: The municipality plants wild edible fruit trees in the yards and premises of residential areas. Only home owners can pick these fruits, so over-picking and disputes are avoided.

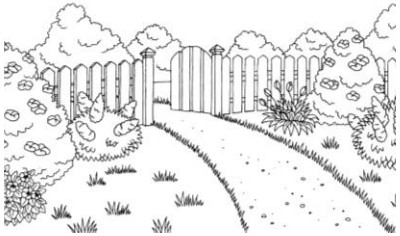

b. Foraging along the way: The municipality plants wild edible fruit trees along street verges, walkways, around schools, public offices, playgrounds and parks. Anyone can pick these fruits, and information about safe and sustainable picking is displayed on the trees.

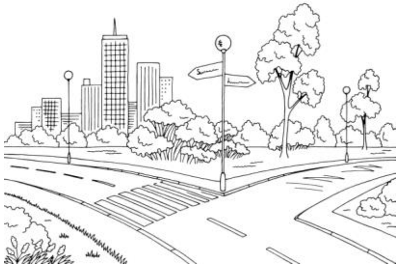

c. Foraging in special parks and gardens: The municipality plants wild edible fruit trees in specific parks and gardens where people can pick them. Anyone can pick fruits in these parks and gardens, provided they follow rules of safe and sustainable picking displayed in the area.

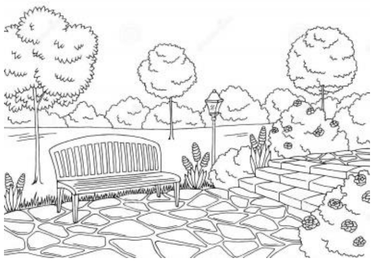

d. Foraging in conservation areas: The municipality allows people to pick wild edible fruits in forests and bush, provided they do not hunt the wildlife or cut trees, and follow rules of safe and sustainable picking. People take permission from the municipality to pick fruits in these areas.

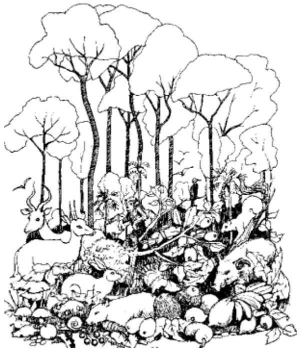

Would municipal authorities allow people to forage in public spaces?

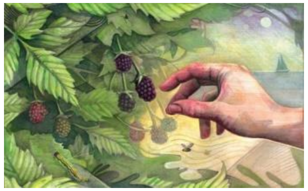

Mostly yes, on the condition that:

1. Foraging is done sustainably, without taking all the fruits
2. Only suitable species and plants that are not threatened are harvested
3. No damage is done to the plants or the surroundings
4. No property is infringed upon, and permission is taken where required

What would the municipal authorities like to know about foraging?

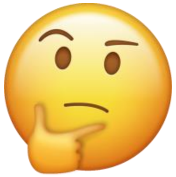

1. Which spaces (verges, parks, bush) they can develop and manage for foragers
2. Which species are suitable for foraging, and how much foraging is sustainable
3. How rules and regulations can be designed and communicated to promote sustainable foraging

Could municipal authorities work together with foragers?

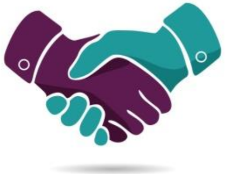

Yes, in a few different ways:

1. Foragers could help the municipality monitor the biodiversity (animals, birds, trees) in the area they forage in
2. Foragers could help the municipality by reporting the waste and invasive aliens in the area they forage in

What are the municipal authorities concerned about in relation to foraging?

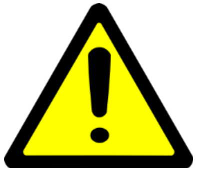

1. Damage to the trees or the surroundings where foraging is undertaken (due to over-picking, trampling, cutting)
2. Disputes between foragers about who can pick and how much can be picked

Would foragers like to see more people foraging in public spaces?

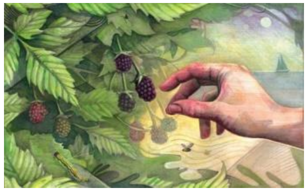

Mostly yes, on the condition that:

1. Foraging is done sustainably, without taking all the fruits
2. No damage is done to the plants or the surroundings
3. No property is infringed upon, and permission is taken where required

What, where and why do people forage?

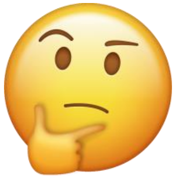

People forage mainly for a number of wild edible fruits, herbs, and sometimes firewood

Many of the people forage in the bush and in their neighbourhoods, and some also forage in vacant lots

Most people forage for leisure and recreation, on their way to work and home, or on the weekends

Could the municipality work together with the foragers?

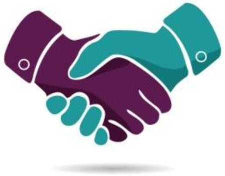

Yes, in a few different ways:

1. By protecting existing foraging areas with rules to prevent logging and over-picking
2. By planting more wild edible fruit trees in public open spaces, including verges, parks, and designated gardens
3. By providing information about rules and sustainable foraging in public spaces

What are the foragers concerned about in relation to foraging?

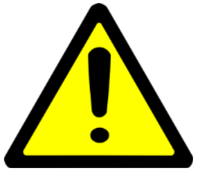

1. Not knowing the legality of foraging in public spaces
2. The threat to foraging spaces from infrastructure development
3. Unsustainable picking by certain individuals
4. Potential disputes among residents and with non-resident foragers
